# Complete genome sequence of *Desulfoscipio* sp. strain L, a putative sulfate-reducing bacterium isolated from contaminated groundwater

**DOI:** 10.1128/mra.00451-25

**Published:** 2025-12-23

**Authors:** Xiaocui Li, Hongyan Wang, Zongming Xiu, Haiwei Wei, Frank E. Löffler, Yi Yang

**Affiliations:** 1 Key Laboratory of Pollution Ecology and Environmental Engineering, Institute of Applied Ecology, Chinese Academy of Sciences 74763 , Shenyang, Liaoning, China; 2 University of Chinese Academy of Sciences 74519, Beijing, China; 3 School of Environment and Geography, Qingdao University 12593, Qingdao, Shandong, China; 4 Department of Civil and Environmental Engineering, University of Tennessee 4292, Knoxville, Tennessee, USA; 5 Department of Biochemistry and Cellular and Molecular Biology, University of Tennessee 4292, Knoxville, Tennessee, USA; 6 Department of Biosystems Engineering and Soil Science, University of Tennessee 4292, Knoxville, Tennessee, USA; 7 Key Laboratory of Forest Ecology and Silviculture, Institute of Applied Ecology, Chinese Academy of Sciences 74763 , Shenyang, Liaoning, China; 8 Shanghai Key Laboratory of Polar Life and Environment Science, Shanghai Jiao Tong University 12474, Shanghai, China; 9 Key Laboratory of Polar Ecosystem and Climate Change, Shanghai Jiao Tong University 12474, Shanghai, China; University of Southern California, Los Angeles, California, USA

**Keywords:** *Desulfoscipio*, sulfate-reducing bacteria

## Abstract

We report the complete genome sequence of *Desulfoscipio* sp. strain L, a putative sulfate-reducing bacterium isolated from contaminated groundwater. The genome comprises a circular chromosome of 4,259,676 bp and a 1,530-bp circular plasmid.

## ANNOUNCEMENT

Members of the genus *Desulfoscipio* are anaerobic chemoorganoheterotrophs capable of utilizing carboxylic acids/alcohols as electron donors, with sulfate, sulfite, or thiosulfate as electron acceptors (1). Currently, only two species are described: *Desulfoscipio gibsoniae* (2) isolated from freshwater mud, and *Desulfoscipio geothermicus* obtained from geothermal groundwater (3). Here, we report the isolation and genomic characterization of *Desulfoscipio* sp. strain L from contaminated groundwater samples collected in April 2012 at an industrial site in Pensacola, Florida, USA (30°25′48″N, 87°13″30″W). Enrichment was performed in bicarbonate-buffered (30 mM) mineral salt medium (4) supplemented with 10 mM lactate and 5 mM sulfate under strict anaerobic conditions. Cultures were incubated statically at 30°C for about 1 month in the dark. Purification was achieved through two rounds of serial dilution-to-extinction (10^−1^ to 10^−10^) in anaerobic medium (4) containing 1% low-melting-temperature agarose, yielding isolated white colonies. The 16S rRNA gene sequence (GenBank PP784321) obtained through colony PCR amplification (primers 27F/1492R (5)) and Sanger sequencing exhibited 95.4% sequence similarity to *D. gibsoniae* DSM 7213 (NR_037081.1) via NCBI BLASTN analysis, below the 98.65% species threshold (6).

To resolve the taxonomic position of strain L and elucidate its metabolic potential, we performed hybrid genome sequencing. For DNA extraction, strain L was cultured anaerobically at 30°C in a liquid bicarbonate-buffered mineral salt medium containing 10 mM lactate and 5 mM sulfate for approximately 1 month in the dark. Cells were harvested at late-exponential phase, and genomic DNA was extracted using the CTAB method (7) and processed for both PacBio and Illumina library preparations. For PacBio sequencing, DNA was sheared to 8–10 kb fragments using g-TUBEs (Covaris, USA), followed by SMRTbell library preparation (SMRTbell Express Template Prep Kit v2.0, Pacific Biosciences) (8). Size-selected libraries were sequenced on a Sequel II System (Chemistry 2.0). For Illumina sequencing, a 350 bp insert library was prepared (NEBNext Ultra DNA Library Prep Kit, New England Biolabs) and sequenced in paired-end mode (2 × 150 bp) on a NovaSeq 6000 platform. Raw reads were quality-controlled using Filtlong v0.2.1 (9) for PacBio data and fastp v0.23.4 (10) for Illumina reads. Hybrid assembly was performed on Galaxy Australia (usegalaxy.org.au) using Unicycler v0.5 (11), incorporating 87,494 PacBio reads (*N*_50_: 15,024 bp) and 4,499,916 Illumina reads. The assembly yielded a complete circular chromosome (4,259,676 bp; 46.8% G+C) (Table 1), verified by overlapping ends. Additionally, a 1,530 bp circular element (66.7% G+C) was identified as a putative plasmid based on its circularity, confirmed by overlapping terminal sequences, and its 70.76% nucleotide sequence similarity to the plasmid of *Bacillus cereus* strain Z4 (Accession: CP130288.1). Genome completeness (97.7%) and contamination (1.5%) were assessed using CheckM v1.2.2 (12). NCBI PGAP annotation (13) identified 3,912 protein-coding genes, 55 tRNAs, 14 rRNAs (5S/16S/23S, 4/5/5), five ncRNAs, and complete sulfate-reduction pathways. Digital DNA-DNA hybridization (dDDH) and ANI analyses performed using the TYGS platform (https://tygs.dsmz.de/) and JSpeciesWS v4.1.1 (14) revealed values of 27.6% dDDH and 83.0% ANI with *D. gibsoniae* DSM 7213, respectively, below established species thresholds. These genomic findings, combined with the 16S rRNA gene sequence divergence, suggest that strain L is a novel *Desulfoscipio* species. The genome provides insights into the metabolic adaptations of sulfate-reducing bacteria in contaminated groundwater ecosystems.

**TABLE 1:** Genome features of strain L

Feature	Data for:	
	Chromosome	Plasmid
Assembly length (bp)	4,259,676	1,530
G+C content (%)	46.8	66.7
No. of assembled contigs	1	1
No. of coding sequences	3912	3
No. of tRNAs	55	0
No. of rRNAs (5S, 16S, 23S)	14 (4, 5, 5)	0
Protein coding genes connected to KEGG pathways	2,018	0
Protein coding genes annotated with COG	3,066	0
Protein coding genes annotated with Pfam	3,132	0
Protein coding genes annotated with Swiss-prot	2,652	0
Protein coding genes annotated with GO	2,577	0
Protein coding genes annotated with NR	3,767	0
GenBank accession	CP156660	CP156661
BioSample accession	SAMN41256859	SAMN41256859
BioProject accession	PRJNA1108883	PRJNA1108883

## Data Availability

The complete genome sequences of *Desulfoscipio* sp. strain L and assembly projects reported in this article have been deposited in DDBJ/ENA/GenBank under the accession numbers listed in Table 1. Raw reads have been deposited in the Sequence Read Archive (SRA) under accession numbers SRR31810663 (Illumina) and SRR31814565 (PacBio).
